# Association between sickle cell disease and the oral health condition of children and adolescents

**DOI:** 10.1186/s12903-018-0629-9

**Published:** 2018-10-20

**Authors:** Carla Figueiredo Brandão, Viviane Maia Barreto Oliveira, Ada Rocha Ramony Martins Santos, Taísa Midlej Martins da Silva, Verônica Queiroz Cruz Vilella, Gleice Glenda Prata Pimentel Simas, Laura Regina Santos Carvalho, Raissa Aires Costa Carvalho, Ana Marice Teixeira Ladeia

**Affiliations:** Bahiana School of Medicine and Public Health, Avenida Dom João VI, no. 275, Brotas. ZIP: 40.290-000, Salvador, Bahia Brazil

**Keywords:** Sickle cell disease, Child, Oral health, Dental caries, Periodontal disease, Saliva

## Abstract

**Background:**

Sickle cell disease (SCD) is the most prevalent monogenic hereditary pathology associated with the presence of hemoglobin SS in the world. It can affect individuals, leading to changes in the face and body, causing a deficiency in dental and bone tissue formation that can ultimately result in a higher level of predisposition to developing dental caries. This study aimed to evaluate the oral condition of children and adolescents with SCD in comparison with the condition of healthy controls.

**Methods:**

This was a cross-sectional study of children and adolescents aged 5 to 18 of both sexes from a hematology center in Bahia, Brazil, and subjects without hemoglobinopathies from a public school of the same state (comparison group). There were 124 individuals, 63 in the comparison group and 61 in the disease group. Interviews, dental and periodontal exams using the DMFT and Periodontal Community Index, respectively, were performed, and the salivary buffer capacity and salivary flow rates of the entire sample population were evaluated. The categorical variables were compared using a chi-square test or Fisher’s exact test. For comparison of means, the Student’s*-t* test was used for independent samples that presented symmetrical distribution.

**Results:**

The study showed that the DMFT was 2.08 (2.71) for the SCD group and 1.05 (1.67) for the comparison group (*p* = 0.013). For dmft, the values were 2.3 (2.6) and 0.88 (1.2), respectively, (*p* = 0.018). Exams of the periodontium showed the presence of gingival bleeding and dental calculus, with no statistical significance between groups (*p* = 0.984). When evaluating salivary flow and buffer capacity, no significant differences were observed for the flow rates (*p* = 0.485), but the SCD group presented a lower buffer capacity compared with the comparison group (*p* = 0.006). Individuals who used hydroxyurea had a dmft (2.50) higher than that of the comparison group (2.00), and salivary flow was lower than the normal rate in 70% of the children who did not use this medication.

**Conclusion:**

Children and teenagers with SCD had deficient oral health when compared with the comparison group, presenting a higher level of dental caries and lower buffer capacity.

## Background

Sickle cell disease (SCD) is caused by a mutant Hb S hemoglobin that modifies a normally shaped cell shape into a sickle shape. This mutant cell defines a group of hemoglobinopathies, at least one of which is Hb S hemoglobin. The most frequent SCD variations are sickle cell anemia (or Hb SS), S beta thalassemia and the heterozygous Hb SC and Hb SD [1].

SCD is a multisystemic disease associated with acute illness and progressive organic damage, leading to organ involvement, which may cause changes in development or functioning. Individuals with this pathology may also present changes in their face, mouth, and teeth caused by the deficient formation of dental and bone tissues [2–4], which may, for example, lead to a higher level of predisposition for developing caries diseases [5–7].

The first study of the prevalence of dental caries in patients with SCD was conducted by Okafor et al. in 1986, who observed a higher prevalence in subjects without SCD (54%) compared with those who had the disease (35.13%) [8]. Since then, some studies have been conducted to verify the association between sickle disease and dental caries, with divergent results [5–7, 9–11]. In the literature, few studies are found that associate the presence of dental caries with salivary flow rates and buffer capacity in sickle cell patients [12]; these are essential factors in the development of caries disease [5–7].

These patients also have a higher risk of developing dental caries due to the high prevalence of opacities in the teeth (alterations in enamel and dentin formation and calcification) [2, 8], frequently used medications containing sucrose and many episodes of hospitalization that make it difficult to perform adequate oral hygiene [13].

Another aspect to be studied is the presence of periodontal problems in sickle cell patients, as these patients are more susceptible to infections. Individuals with SCD have a higher level of predisposition to developing periodontal disease due to the presence of these pathogens in the mouth, as well as alterations in their cellular and humoral immune response [14, 15].

From the knowledge of how these diseases manifest, health promotion measures can be adopted and target treatments instituted for this group of patients, respecting their individualities.

This study aimed to evaluate the oral condition of children and teenagers diagnosed with SCD compared with healthy controls and to assess the influence of salivary flow and buffer capacity on predicting oral health.

## Methods

This was a descriptive and analytical cross-sectional study conducted with children and teenagers aged 5 to 18 of both sexes, from the Hematology and Hemotherapy Foundation in Bahia (HEMOBA), who formed the sickle cell disease group. They were compared with subjects without hemoglobinopathies, selected from among children enrolled in the State School Francisco da Conceição Menezes (comparison group). To be selected for the SCD group, the following inclusion criteria were to be met: Participants had to have SCD and Hb S diagnosed by Hb electrophoresis and/or high-performance liquid chromatography. For the comparison group, individuals had to be in the same age group, not have SCD and be clinically healthy. In both groups, participants were not to be undergoing orthodontic treatment. This research was approved by the Research Ethics Committee of the Bahiana School of Medicine and Public Health and is referenced as 54.637.816.7.0000.5544. The participants’ signature of the term of informed consent/assent ensured the possibility of conducting the study.

Our sample was calculated by estimating that the prevalence of oral disorders in the general population would be 50%; expecting to find a detectable difference of 20% in the prevalence of the group with SCD for a value of α = 0.05, a minimum of 48 individuals in each group was necessary.

### Data collection

In this research, the dental data were collected in accordance with the methodology used by the SB Brasil Project Field Team 2010 and recommended by the World Health Organization (WHO) [16]. Data consisted of records of the teeth, obtained by using mean dmft indexes for deciduous dentition and DMFT for permanent dentition, and reported as the sum of decayed, missing and filled teeth [17].

The Periodontal Community Index (CPI), an instrument used by the SB Brasil 2010 Project, was used to examine the periodontium according to the field team manual [17], which allowed evaluation of periodontal health relative to hygiene, gingival bleeding and the presence of calculus in children over 12 years of age. In each sextant of the mouth - 16, 11, 26, 36, 31 and 46, six teeth were examined on each of the buccal and lingual surfaces, covering the mesial, middle and distal regions.

When there was no index tooth, the sextant was canceled [17]. This evaluation was performed only in patients older than 12 years; this is an international standard for assessing the conditions of tooth injuries, since it is the youngest age at which the individual has the complete permanent dentition, disregarding eruption of the third molar [18].

Subjects under 18 years of age were not treated; thus, the presence of a periodontal pocket was not investigated since soft tissue alterations could be associated with the eruption of a tooth and not with the presence of pathological periodontal changes [17].

A clinical file was adapted from epidemiological studies conducted by the WHO [16], in the fourth edition of oral health surveys. These basic methods guided the data necessary for characterization of the sample, which were recorded individually among participants and included name, age, and sex. The following information was added: skin color (self-reported), schooling, family income, time of diagnosis of the disease, use of medications, daily oral hygiene and regularity of attendance at dental appointments (provided by the dentist), as well as saliva conditions.

The calibration exercise was carried out in two steps. In the first step, a single investigator conducted theoretical training for recognition of the different oral health conditions according to the SB Brasil 2010 Field Team Manual and to standardize the oral exams and the diagnostic criteria [17]. The next step consisted of the examination of 12 children and adolescents. The exams were performed on two separate occasions with a 4-month interval between sessions. Data analysis involved the calculation of Kappa coefficients for the evaluation of intra-observer agreement. (Kappa = 0.71).

During the examination, the child was seated in a chair, under natural light, and the examiner used a flat mouth mirror, a CPI probe for the oral epidemiological examination, gauze and a wooden spatula [17].

To determine the salivary flow rate and assess the amount of saliva produced by the child in 1 min, a piece of paraffin (Parafilm ®), a 25 mL graduated beaker, a funnel and a stopwatch were used. First, the paraffin was left in the patient’s mouth for 1 min; the patient was asked to swallow the accumulated saliva during that period; then Parafilm® was chewed for 5 min [19, 20]. After this, the saliva produced was collected through a funnel into a beaker from the time the stopwatch was started, and the salivary flow was measured directly by reading the total volume of stimulated saliva obtained in the time determined. The final result was expressed in milliliters of stimulated saliva produced per minute (mL/min). The results were interpreted by the amount of saliva produced: normal (above 1.0 mL/min), low (0.7 to 1.0 mL/min), very low (0.1 to 0.7 mL/min), xerostomia (below 0.1 mL min) and hypersalivation (above 2.00 mL/min) [19].

For the buffer capacity, 1.0 mL of the saliva collected from each child was added to 3.0 mL of 0.005% HCl solution. After stirring the tube and waiting for 10 min, this mixture was taken for pH reading in the digital potentiometer DMPH-2, which was previously calibrated using pH 4.0 and 7.0. The buffer capacity was expressed by the potentiometer reading of the final pH of the saliva-acid mixture and evaluated as normal (above 6.0), reduced (5.5) and low (below 4. 0) [19].

### Statistical analysis

Statistical analysis was then performed with the descriptive measures. Quantitative variables were represented by their means and standard deviations. Categorical variables were expressed by frequencies and percentages. For comparison of the categorical variables, the Chi-square test was used through bivariate analysis. The Student’s t-test for independent samples was used to compare the means. The Statistical Package for the Social Sciences (SPSS Inc., Chicago, IL, United States of America), version 21.0 was used.

## Results

A total of 124 children and teenagers were examined, of whom 61 had SCD and 63 were healthy. Their distribution by age, sex, race (self-reported), educational level of the child and mother, as well as family income are described in Table 1, and no difference was observed between groups.

**Table 1:** Socio-demographic characteristics of the sample

	SCD Group (*n* = 61)	Comparison Group (*n* = 63)	*p* value
Age (years) mean ± SD	12.4(2.9)	11.1 (2.9)	0.014*
Age Range			0.119**
5 to 8 years	6 (9.8)	11 (17.5)	
9 to 12 years	24 (39.3)	31 (49.2)	
13 to 18 years	31 (50.8)	21 (33.3)	
Sex			0.605**
Male	34 (55.7)	38 (60.3)	
Female	27(44.3)	25 (39.7)	
Race			0.066**
Black	18 (29.5)	26 (41.3)	
Mixed	42 (69.9)	32 (50.8)	
Others	1 (1.6)	5 (8)	
Educational Level	(*n* = 60)	(n = 61)	0.114**
Illiterate	4 (6.7)	3 (4.9)	
Middle school	48 (80.0)	56 (91.8)	
High school	8 (13.3)	2 (3.3)	
Maternal Education	(*n* = 56)	(n = 56)	0.902**
Illiterate	2 (3.6)	1 (1.8)	
Middle school	28 (50.0)	31 (55.4)	
High school	23 (41.1)	21 (37.5)	
University	3 (5.4)	3 (5.4)	
Income	(*n* = 57)	(n = 55)	0.081**
No income	8 (14.0)	3 (5.5)	
Up to 1 monthly Brazilian minimum wage	40 (70.2)	35 (63.6)	
Above 2 monthly Brazilian minimum wages	9 (15.8)	17 (30.9)	

* Independent t-test; ** Chi-square test; m = average; SD = standard deviation. Brazilian minimum wage = US$300.00. *p* < 0.05

DMFT and dmft data are described in Tables 2 and 3. These indexes were higher for the SCD group than for the comparison group and were statistically significant. The decayed component was found to be more predominant in the permanent and deciduous dentition of the SCD group.

**Table 2:** Means of DMFT values of children and adolescents

	SCD Group (*n* = 61)	Comparison Group (*n* = 63)	*p* value
Dental Condition	mean ± SD	mean ± SD	
Decayed	1.5 ± 2.38	0.8 ± 1.23	0.003*
Missing	0.8 ± 0.33	0.98 ± 0.3	0.620*
Filled	0.5 ± 1.0	0.24 ± 0.7	0.004*
DMFT	2. ± 2.7	1.1 ± 1.7	0.013*

*Independent t-test; SD = standard deviation. DMFT - mean of the components D (decayed), M (missing or extracted due to caries) and F (filled or restored) permanent dentition. *p* < 0.05

**Table 3:** Means of dmft values in children assessed

	SCD Group (*n* = 23)	Comparison Group (*n* = 24)	*p* value
Dental Condition	mean ± DP	mean ± DP	
Decayed	1.3 ± 1.8	0.7 ± 1.12	0.039*
Missing/Extracted	0.65 ± 1.23	0.12 ± 0.34	0.000*
Filled	0.35 ± 0.8	0.2 ± 0.6	0.222*
dmft	2.3 ± 2.6	0.88 ± 1.2	0.018*

*Independent t-test; SD = standard deviation. Dmft - Mean of the components d (decayed), and m (extracted) and f (filled or restored) deciduous dentition. *p* < 0,05

There was no association between SCD and caries disease in this study. Decayed teeth were found in 36 subjects of the SCD group (59%) and in 30 subjects (49.2%) of the comparison group (*p* = 0.276).

The results of the periodontal examination were classified according to their degree of involvement, but no difference was observed between the groups (Table 4).

**Table 4:** Mean and percentage values of individuals according to their periodontal condition

	SCD Group (*n* = 35)	Comparison Group (*n* = 37)	*p* value
Periodontal Condition	n (%)	n (%)	0.984**
Healthy periodontium	1 (2.9)	1 (2.7)	
Gingival bleeding	12 (34.3)	12 (32.4)	
Dental calculus	22 (62.9)	24 (64.9)	

** Chi-square test. *p* < 0.05

As regards the salivary examination, the results are described in Table 5. In the majority of the sample, the buffer capacity of saliva was evaluated as being normal. In the SCD group, 83.1% of its subjects had normal salivary pH and 17% had reduced pH. On the other hand, in the comparison group, 98.2% of the participants had a pH within the normal range. Statistically significant differences were found between groups when assessing reduced buffer capacity (*p* = 0.006).

**Table 5:** Sample distribution according to salivary conditions

	SCD Group *n* = 59 (%)	Comparison Group *n* = 55(%)	*p* value
Salivary Flow			0.485**
Hyposalivation (0.1 to 0.7 mL/min)	19 (32.2)	18 (32.7)	
Low (0.70 to 1.0 mL/min)	17 (28.8)	13 (23.6)	
Normal (> 1.0 mL/min)	23 (39.0)	22 (40.0)	
Hypersalivation (> 2.0 mL/min)	0	2 (3.6)	
Buffer Capacity			0.006**
Normal (pH > 6.0)	49 (83.1)	54 (98.2)	
Reduced (below 5.5)	10 (16.9)	1 (1.8)	

** Chi-square test. *p* < 0.05

The salivary flow presented no significant difference between the groups since the distribution between them was similar (*p* = 0.485).

A relationship was found between DMFT and buffer capacity in patients with SCD, and there was a significant difference between the groups in the decayed component and DMFT; however, this value was higher in participants in the SCD group who had normal buffer capacity (Table 6).

**Table 6:** Comparison of the mean DMFT with the buffer capacity of children and adolescents with SCD

	Normal (*n* = 49)	Reduced (*n* = 10)	*p* value
Dental Condition	mean ± SD	mean ± SD	
Decayed	1.5 ± 1.99	0.5 ± 0.84	0.014*
Missing	0.8 ± 0.33	0.10 ± 0.3	0.877*
Filled	0.6 ± 1.2	0.20 ± 0.42	0.063*
DMFT	2.20 ± 2.4	0.6 ± 1.0	0.002*

* Independent t-test; SD = standard deviation. *p* < 0.05

Table 7 shows the results for individuals with SCD in regard to use of hydroxyurea; 33 (54.1%) of these patients took this medication, and 28 (45.9%) patients did not. When assessing the association between DMFT and hydroxyurea, individuals who did not use the medication were observed to have a higher DMFT value. However, in deciduous dentition, dmft values were higher for those who had taken the medication, and the number of filled teeth was also higher, but there was a significant difference (Table 8).

**Table 7:** Comparison of the mean DMFT values of children and adolescents with Hb SS relative to the use of hydroxyurea

	Users of Hydroxyurea(*n* = 33)	Non users of Hydroxyurea(*n* = 28)	*p* value
Dental Condition	mean ± SD	mean ± SD	
Decayed	1.12 ± 1.78	1.96 ± 2.91	*0.122
Missing	0.90 ± 0.29	0.07 0.37	*0.718
Filled	0.45 ± 0.90	0.60 ± 1.28	*0.121
DMFT	1.64 ± 2.11	2.61 ± 3.23	*0.024

* Independent T-test; SD = standard deviation. *p* < 0.05

**Table 8:** Comparison of the mean dmft values of children and adolescents with Hb SS relative to the use of hydroxyurea

	Users of Hydroxyurea (*n* = 14)	Non-users of Hydroxyurea (*n* = 9)	*p* value
Dental Condition	m ± SD	m ± SD	
Decayed	1.35 ± 1.94	1.22 ± 1.71	*0.913
Extracted	0.64 ± 1.39	0.66 ± 1.00	*0.934
Filled	0.50 ± 0.94	0.11 ± 0.33	*0.021
dmft	2.50 ± 2.95	2.00 ± 1.93	*0.149

* Independent t-test; SD = standard deviation. *p* < 0.05

Table 9 presents the findings of salivary conditions in individuals who did and did not use hydroxyurea. The salivary flow was lower than the normal rate in 75% of children and adolescents who used the medication. The buffer capacity was reduced in 18.2% of the medication users.

**Table 9:** Sample distribution according to salivary conditions and hydroxyurea

	Users of Hydroxyurea (*n* = 33)	Non users of Hydroxyurea (*n* = 26)	*p* value
Salivary Flow			0.032**
Hyposalivation (0.1 to 0.7 mL/min)	13 (39.4)	6 (23.1)	
Low (0.7 to 1.0 mL/min)	12 (36.4)	5 (19.2)	
Normal (> 1.0 mL/min)	8 (24.2)	15 (57.7)	
Hypersalivation (> 2.0 mL/min)	0	0	
Buffer Capacity			0.776**
Normal (pH > 6.0)	27 (81.8)	22 (84.6)	
Reduced (below 5.5)	6 (18.2)	4 (15.4)	

** Chi-square test. *p* < 0.05

## Discussion

SCD is a disease that has been widely discussed in several ways that are important for understanding it. This recessive and hereditary disease was discovered in African black people; however, today it affects individuals with other racial characteristics because of intermarriage [21]. In this study, there was a higher prevalence of brown individuals, although Bahia is a state with one of largest black populations in Brazil and an extensive mixture of races.

The consequences of SCD to the human body have been explored from multiple aspects. Among these observations, there has been rising concern about its manifestations in some parts of the oral cavity, which causes alterations in different tissues that form teeth and bones, in spite of these manifestations not being pathognomonic signs of the disease [3, 4, 9, 22, 23].

Caries disease is a globally studied pathology; its emergence and development are related to intrinsic factors associated with socioeconomic, cultural and educational aspects that are demonstrated by the degree of impairment of the oral health of the affected population [9, 24, 25]. In this study, all of the abovementioned factors were constantly present in all the subjects; therefore, these could not be considered potential confounding biases.

No association of caries disease with SCD was observed in this study because the presence of decayed teeth was verified in 59% of the individuals in the SCD group, and in 49.2% of the comparison group; that is, there was a difference of 10% between them. The data in this research corroborated the findings of the study by Passos et al. [10], who assessed 190 patients of African descent, with and without SCD; these patients had a mean age of 30, which was higher than in our study (11.7 years). Luna et al. [26] found a prevalence of 47% of caries in 250 children and adolescents with SCD, a lower value than that found in this study.

Nevertheless, some studies have found this association when evaluating different age groups. Luna et al. [9] evaluated 160 children with SCD aged 3 to 12 years of age in Recife and found a higher frequency of dental caries in children with SCD. Similarly, Laurence et al. [5] conducted a retrospective cohort study in Baltimore and Washington with 102 individuals over 18 years, with and without SCD. They also found an association when they assessed the presence of caries disease and socioeconomic factors; they verified that individuals with SCD coming from lower-income families had a greater tendency towards caries disease because they had less access to treatment.

Furthermore, in this study, the DMFT and dmft values found were 2.1 and 2.30, respectively, for individuals with SCD and 1.1 and 0.88 for the comparison group. This result was close to the values in the study by Luna et al. [9] that evaluated only children with SCD and found a DMFT of 1.5 and dmft of 2.2, mainly for deciduous teeth. Furthermore, Fernandes et al. [7] analyzed 56 children and 50 adolescents with SCD and 205 children and 180 adolescents in a control group, both aged 8 to 14, at a hematology center in Minas Gerais, Brazil. In this case, the DMFT value was 1.3 in the SCD group and 1.8 in the control group. For patients with SCD, in general, the condition of their oral health was better.

In our study, in spite of the SCD subjects having worse oral health conditions, the observed results agreed with the SB Brasil 2010 [27] data, which found dmft = 2.43 and DMFT = 2.07.

In a similar manner, Ralstrom et al. [11] evaluated 54 American adolescents of African descent with a mean age of 14 years, who had the Hb SS and Hb SC sickle cell genotypes. The mean DMFT found was 1.94 in the disease group and 2.96 in the control group. There was no significant difference in the frequency values for dental caries between the SCD adolescents and controls, potentially due to high exposure to the fluoride present in the water supply in that region. The data found were similar to those of the present study relative to individuals with SCD. It is worth noting that in Brazil there are public health policies that regulate the fluoridation of the water supply; this is an important measure for the prevention of caries disease [28].

In the study conducted by Singh et al. [6] in India, with 750 patients with SCD and Betalassemia, aged 3 to 15 years, a DMFT of 6.59 was observed for patients with the disease. This value differs from our findings and those of other studies, probably due to different health policies in the regions concerned, possibly making treatment less accessible.

Costa et al. [29], when evaluating the care offered to patients with SCD in Maranhão, Brazil, found that children had fewer filled teeth than did adults and that there was also an increase in number of filled teeth as age increased. Dental treatments were probably due to the lack of specific oral health programs for this population. These patients often present severe systemic health problems that can place their lives at risk. Their oral health care is neglected, and they are denied access to preventive care; thus, only curative treatment programs are available to them [6, 29]. These factors were probably responsible for the increase in caries in the population of this study.

The presence of caries disease, a lack of attention to the need, and a worsening of the condition lead to the need for more complex treatments. This need is not only limited to the population studied but also affects the general population with similar socio-demographic characteristics, as was reflected in the data of SB Brasil 2010 [6, 11, 27].

With regard to the periodontal condition, the presence of SCD did not change its oral manifestation. Individuals aged 12 years and older presented a similar situation with the presence of gingival bleeding, and the majority of them had dental calculus. Passos et al. [10] also found no association between sickle cell disease and periodontal disease when evaluating 190 patients, 99 with systemic alteration and 91 controls. Fernandes et al. [7] found that only the adolescents showed the presence of gingival bleeding, but no significant differences were observed between the SCD and control groups. Carvalho et al. [30] evaluated several criteria indicative of periodontal diseases in patients with SCD, patients with the trait of the disease and patients without the disease. They observed that none of these criteria were associated with the patients with SCD, suggesting no association between these two pathologies.

Mahmoud, Ghandour and Atalla [31] evaluated the association between periodontal disease and SCD in 113 adolescents aged 12 to 16 and found no statistically significant differences between the groups; but when evaluating the disease group, they were able to verify an increase in the prevalence of gingival inflammation in adolescents with SCD when compared with the control group. Tonguç, Unal and Aspaci [32] also verified a lack of differences in the periodontal health status of 49 children with SCD and 39 systemically healthy children in the control groups. The most important finding of their study was that gingival enlargement was more prevalent in children with SCD. Singh et al. [6], in their study, observed a higher prevalence of periodontal disease in patients with beta thalassemia, followed by those with SCD, and, last, the controls.

Salivary flow is an essential and reliable measure to evaluate pathological alterations [33–35]. Reduced salivary flow may cause greater vulnerability to caries disease and oral infections and to changes in chewing, swallowing, tasting and speaking [36]. When the saliva is stimulated, it may promote positive actions in the oral cavity, such as potentiation of tooth remineralization capacity, removal of substances, neutralization of acids and antimicrobial action [37].

Studies have shown a positive correlation between salivary flow and the buffer capacity of saliva [21, 38]. Few articles were found on the topic of salivary parameters in children and adolescents with SCD. In our study, the observed salivary flow was lower in 61% of sickle cell patients and in 56.3% of patients the comparison group.

Leone et al. [14] carried out a systematic review of 600 articles in the MEDLINE and EMBASE databases on salivary aspects as indicators of caries disease risk and concluded that the salivary buffer capacity presented a weak-to-moderate association with the risk of developing the disease, unlike the flow that showed a strong correlation with its appearance. This study showed a prevalence of 17% for reduced buffer capacity in the SCD group. A decrease in salivary flow was found in both groups, suggesting that the association of these factors may lead to higher predisposition for the development of dental caries. Furthermore, the SCD group had a higher risk of developing caries.

It is interesting to notice that in our study, among individuals with SCD, those with normal saliva buffer capacity had higher DMFT values than those with reduced buffer capacity; this difference was specifically observed relative to the number of decayed teeth. Buffer capacity and salivary flow alone cannot be used as determinant indexes for diagnosis of caries disease since other factors need to be considered to determine the potential of cariogenic activity. Bacterial biofilms, deficient oral hygiene, systemic diseases, previous and/or current use of a fluoridated water supply, the frequency of sugar ingestion and microorganism counts contribute to dental caries development [1, 2, 5, 8–12]. The continuous use of medications may cause xerostomia and thus increase the risk of caries disease development, as they lead to a decrease in salivary flow and cause changes in saliva [36].

Hydroxyurea, which is approved by the US Food and Drug Administration (FDA), is a drug currently used in the treatment of Hb SS [39]. This drug has a strong positive impact on the quality of life of SCD patients by reducing many negative aspects of the disease including vaso-occlusive crises, the need for transfusions, the number of hospitalizations, the length of hospital stays, and acute neurological events; in addition, it has decisively demonstrated a reduction in the number of deaths resulting from neurological events or SCD when compared with the same number of patients in a group not using the drug. [40]

Salvia et al. [41] assessed 69 patients with a mean age of 26 years who had SCD. Among these patients, there were users and non-users of hydroxyurea. When evaluating salivary flow and DMFT, these authors verified that DMFT was higher in patients taking the medication (9.10 ± 6.93) than in those who did not or in the respective controls (7.67 ± 6.06; 7.72 ± 5.91; 7.59 ± 7.14). All groups presented normal salivary flow, ranging from 1.21 ± 0.88 to 1.33 ± 0.73. In our study, 32 individuals used hydroxyurea, and the DMFT and dmft results were lower for children and adolescents using the medication (1.64 ± 2.11; 2.0 ± 2.95). When evaluating the saliva, 75% of those taking the medication had low salivary flow and 18% had reduced buffer capacity. This divergence in results may be related to differences in the methodology.

No other studies about hydroxyurea and its effects on the oral cavity were found in the researched literature, but this study showed that the use of the medication changed the salivary flow and buffer capacity of saliva, which predisposed patients to the development of oral pathologies.

Another aspect that should be discussed in this study was that both groups evaluated were mostly treated mostly in the Sistema Único de Saúde – SUS, a public health care program that offers patients basic care without adopting an effective health promotion plan. At this health center, patients with SCD are offered routine medical appointments, administration of medications, transfusions, and referrals for hospitalization in the most severe cases. However, due to the lack of specialized care in the different areas of dentistry, such as pediatric dentistry, endodontics, prosthesis, periodontics and orthodontics, the patients who demand/require these specific treatments are not always given the appropriate attention.

In Brazil and in other countries it has been observed that in the most severe cases of the disease, the patients who are most affected systemically require hospitalizations for transfusions and treatments. These patients require primary oral health care, such as daily tooth brushing after main meals and careful selection of the type of diet consumed, both of which may lead to a lower level of predisposition for developing oral diseases [5, 8, 10].

There were some limitations to this study. The examiner was not blinded to the dental examination and, therefore, could have introduced examiner’s bias. However, this aspect was calibrated for in the field data collection phase, and the clinical criteria were clearly defined; thus, this bias is unlikely to have distorted the data from the exams. A few of the more severely ill SCD patients were unwilling to participate because of the discomfort caused by their disease symptoms. Perhaps this could have minimized the differences between the groups.

## Conclusion

The children and adolescents with SCD had unfavorable oral conditions when compared with healthy patients, presenting higher dental caries indexes in both deciduous and permanent dentition and lower buffer capacity values. These results suggested that these individuals needed better and continuous oral health care integrated with the clinical aspects of their systemic health.

## Abbreviations

CPI: Periodontal Community Index
dmft: mean of the components d (decayed), and m (extracted) and f (filled or restored) deciduous dentition
DMFT: mean of the components D (decayed), M (missing or extracted due to caries) and F (filled or restored) permanent dentition
DNA: deoxyribonucleic acid
Hb SC: Genotype hemoglobin SC
Hb SD: genotype hemoglobin SD
Hb SS: genotype hemoglobin SS
HCL: hydrochloric acid
min: minute
mL: milliliter
SCD: sickle cell disease
WHO: World Health Organization

## References

[1] Brasil. Ministério da Saúde. Secretaria de Atenção à Saúde. Departamento de Atenção à Saúde. Doença Falciforme: Condutas básicas para tratamento**.** Brasília (DF), Ministério da Saúde, 2012. (Série B. Textos Básicos de Saúde).

[2] Taylor LB, Nowak AJ, Giller RH, Casamassimo PS (1995). Sickle cell anemia: a review of the dental concerns and a retrospective study of dental and bony changes. Spec Care Dentist.

[3] Souza SFC, HLCC C, CPS C, EBAF T. Association of sickle cell haemoglobinopathies with dental and jaw bone abnormalities. Oral Dis. 2017. 10.1111/odi.12742 28833998

[4] Carvalho HLCC, Rolim JYS, Thomaz EBAF, Souza SFC (2017). Are dental and jaw changes more prevalent in a Brazilian population with sickle cell anemia?. Oral Surg Oral Med Oral Pathol Oral Radiol.

[5] Laurence B, George D, Woods D, Shosanya A, Katz RV, Lanzkron S (2006). The association between sickle cell disease and dental caries in African Americans. Spec Care Dentist..

[6] Singh J, Singh N, Kumar A, Kedia NB, Agarwal A (2013). Dental and periodontal health status of Beta thalassemia major and sickle cell anemic patients: a comparative study. J Int Oral Health.

[7] Fernandes MLMF, Kawachi I, Fernandes AF, Corrêa-Faria P, Paiva SM, Pordeus IA (2016). Caries prevalence and impact on oral health-related quality of life in children with sickle cell disease: cross-sectional study. BMC Oral Health.

[8] Okafor LA, Nonnoo DC, Ojehanon PI, Aikhionbare O (1986). Oral and dental complications of sickle cell disease in Nigerians. Angiology.

[9] Luna AC, Rodrigues MJ, Menezes VA, Marques KM, Santos FA (2012). Caries prevalence and socioeconomic factors in children with sickle cell anemia. Braz Oral Res.

[10] Passos CP, Santos PRB, Aguiar MRC, Cangussu MC, Toralles MB, da Silva MC (2012). Sickle cell disease does not predispose to caries or periodontal disease. Spec Care Dentist..

[11] Ralstrom E, da Fonseca MA, Rhodes M, Amini H. The impact of sickle cell disease on oral health-related quality of life. Pediatr Dent 2014; 36:24–28. 24717705

[12] Leone CW, Oppenheim FG (2001). Physical and chemical aspects of saliva as indicators of risk for dental caries in humans. J Dent Educ.

[13] Brasil. Ministério da Saúde. Secretaria de Atenção à Saúde. Departamento de Atenção Especializada. Manual de Educação em Saúde. Brasília (DF): Ministério da Saúde; 2008.

[14] Javed F, Correa FOB, Nooh N, Almas K, Romanos GE, Al-Hezaimi K (2011). Orofacial manifestations in patients with sickle cell disease. Am J Med Sci.

[15] Veiga PC, Schroth RJ, Guedes R, Freire SM, Nogueira-Filho G (2013). Serum cytokine profile among Brazilian children of African descent with periodontal inflammation and sickle cell anemia. Arch Oral Biol.

[16] World Health Organization (1997). Oral health surveys: basic methods.

[17] Brasil. Secretaria de Vigilância à Saúde. Secretaria de Atenção à Saúde. Departamento de Atenção Básica. Coordenação Nacional de Saúde Bucal. SB Brasil 2010: Manual da Equipe de Campo**.** Brasília (DF). 2009; 37–42.

[18] Domingos PAS (2010). Aspectos epidemiológicos da saúde bucal de crianças em um município brasileiro. Arq Odontol.

[19] Arai OS, Camargo ALS, Jorge AOC, Rego MA (2003). Avaliação do risco de cárie em crianças através de método convencional e do programa cariograma. JBP J Bras Odontopediatr Odontol Bebê.

[20] Garcia LB, Bulla JR, Kotaca CR, Tognim MCB, Cardoso CL (2009). Testes salivares e bacteriológicos para avaliação do risco de cárie. RBAC.

[21] Calvo-Gonzalez E, Rocha V (2010). “Está no sangue”: a articulação de ideias sobre“raça”, aparência e ancestralidade entre famílias de portadores de doença falciforme em Salvador. Bahia Revista de Antropologia.

[22] Acharya S (2015). Oral and dental considerations in management of sickle cell anemia. Int J Clin Pediatr Dent.

[23] Botelho DS, Vergne AA, Bittencourt S, Ribeiro EP (2009). Perfil sistêmico e conduta odontológica em pacientes com anemia falciforme. Int J Dent.

[24] Berkowitz RJ (2003). Causes, treatment and prevention of early childhood caries: a microbiologic perspective. J Can Dent Assoc.

[25] Çolak H, Dulgergil ÇT, Dalil M, Hamidiet MM (2013). Early childhood caries update: a review of causes, diagnoses and treatments. J Nat Sci Biol Med.

[26] Luna A, Gomes M, Granville-Garcia A, Menezes V (2018). Perception of treatment needs and use of dental Services for Children and Adolescents with sickle cell disease. Oral Health Prev Dent.

[27] Brasil. Ministério da Saúde. Coordenação Nacional de Saúde Bucal. Projeto SB Brasil 2010: Pesquisa Nacional de Saúde Bucal: resultados principais. Brasília (DF), 2011.

[28] Ramires I, Buzalaf MAR (2007). A fluoretação da água de abastecimento público e seus benefícios no controle da cárie dentária: cinquenta anos no Brasil. Ciênc Saúde Coletiva.

[29] Costa SPC, Aires BTC, Thomaz EBAF, Souza SFC (2016). Dental care provided to sickle cell anemia patients stratified by age: a population-based study in northeastern Brazil. Eur J Dent.

[30] Carvalho HLCC, Thomaz EBAF, Alves CMC, Souza SFC. Are sickle cell anemia and sickle cell trait predictive factors for periodontal disease? A cohort study. J Periodontal Res. 2015:1–15. 10.1111/jre.12342 26670655

[31] Mahmoud MO, Ghandour IA, Atalla B (2013). Association between sickle cell anemia and periodontal disease among 12 to 16 year old Sudanese children. Periodontal disease. Oral Health Prev Dent.

[32] Tonguç MO, Unal S, Aspaci RB (2018). Gingival enlargement in children with sickle cell disease. J Oral Sci.

[33] Moimaz SAS, Garbin CAS, Aguiar ACA, Silva MB (2002). Capacidade Tampão da Saliva Frente a Diversos Estímulos Gustativos. Rev Fac Odontol Lins.

[34] Bretas LP, Rocha ME, Vieira MS, Rodrigues ACP (2008). Fluxo salivar e capacidade tamponante da saliva. Pesqui Bras Odontopediatria Clín Integr.

[35] Cortelli SC, Chaves MGAM, Faria IS, Landucci LF, Oliveira LD, Sherma AP (2002). Avaliação da condição bucal e do risco de cárie de alunos ingressantes em curso de Odontologia. PGR-Pós-Grad rev.

[36] World Health Organization. Oral Health Surveys Basic Methods. 5th ed. Brazil: World Health Organization; 2013.

[37] Tenovuo J (2002). Antimicrobial agents in saliva — protection for the whole body. J Dent Res.

[38] Krasse B (1988). Exame da saliva. Risco de cárie: guia prático para controle e assessoramento.

[39] Ware RE, Aygun B (2009). Advances in the use of hydroxyurea. Hematol Am Soc Hematol Educ Program.

[40] Cançado RD, Lobo C, Angulo IL, Araújo PCI, Jesus JA (2009). Clinical protocol and therapeutic guidelines for the use of hydroxyurea in sickle cell disease. Revista Brasileira de Hematologia e Hematerapia.

[41] Salvia ARD, Figueiredo MS, Braga JAP, Pereira DFA, Brighenti FL, Koga-Ito CY (2013). Hydroxyurea therapy in sickle cell anemia patients aids to maintain oral fungal colonization balance. J Oral Pathol Med.

